# Functional enrichment analyses and construction of functional similarity networks with high confidence function prediction by PFP

**DOI:** 10.1186/1471-2105-11-265

**Published:** 2010-05-19

**Authors:** Troy Hawkins, Meghana Chitale, Daisuke Kihara

**Affiliations:** 1 Department of Medical and Molecular Genetics, Indiana University School of Medicine, R3-C660, 980 W Walnut St., Indianapolis, IN 46202, USA; 2 Department of Computer Science, College of Science, Purdue University, West Lafayette, IN 47907, USA; 3 Department of Biological Sciences, College of Science, Purdue University, West Lafayette, IN 47907, USA; 4 Markey Center for Structural Biology, College of Science, Purdue University, West Lafayette, IN, 47907, USA

## Abstract

**Background:**

A new paradigm of biological investigation takes advantage of technologies that produce large high throughput datasets, including genome sequences, interactions of proteins, and gene expression. The ability of biologists to analyze and interpret such data relies on functional annotation of the included proteins, but even in highly characterized organisms many proteins can lack the functional evidence necessary to infer their biological relevance.

**Results:**

Here we have applied high confidence function predictions from our automated prediction system, PFP, to three genome sequences, *Escherichia coli*, *Saccharomyces cerevisiae*, and *Plasmodium falciparum *(malaria). The number of annotated genes is increased by PFP to over 90% for all of the genomes. Using the large coverage of the function annotation, we introduced the functional similarity networks which represent the functional space of the proteomes. Four different functional similarity networks are constructed for each proteome, one each by considering similarity in a single Gene Ontology (GO) category, *i.e. *Biological Process, Cellular Component, and Molecular Function, and another one by considering overall similarity with the *funSim *score. The functional similarity networks are shown to have higher modularity than the protein-protein interaction network. Moreover, the *funSim *score network is distinct from the single GO-score networks by showing a higher clustering degree exponent value and thus has a higher tendency to be hierarchical. In addition, examining function assignments to the protein-protein interaction network and local regions of genomes has identified numerous cases where subnetworks or local regions have functionally coherent proteins. These results will help interpreting interactions of proteins and gene orders in a genome. Several examples of both analyses are highlighted.

**Conclusion:**

The analyses demonstrate that applying high confidence predictions from PFP can have a significant impact on a researchers' ability to interpret the immense biological data that are being generated today. The newly introduced functional similarity networks of the three organisms show different network properties as compared with the protein-protein interaction networks.

## Background

The recent paradigm shift in molecular and systems biology to characterization of large sets of genes and proteins has been enabled by continual technological innovations, including fast sequencing technologies [1-3], arrays for measuring gene expression patterns [4], and high throughput screens that identify various types of molecular interactions [5-7]. Data sets produced by these new technologies have also spurred development of computational tools to assist in their analysis [8-10]. Of particular importance is function assignment to genes in a genome or any system of interest, as functional information is indispensable for both biological interpretation of the behavior of the system and generation of hypotheses for designing subsequent experiments [11]. To this end, many function prediction methods have been developed recently to meet the urgent needs [12]. They include those which employ information from sequence database search [13-17] more thoroughly than conventional homology searches [18,19], those which use protein tertiary structure information [20-23], methods that consider conservation of gene locations in genome sequences [24,25], and methods which utilize protein-protein interaction (PPI) data [26-28]. Please refer to recent reviews for thorough discussion of recent function prediction methods [9,29].

We previously introduced PFP as a method for predicting Gene Ontology (GO) functional terms [30] for individual protein sequences with empirically derived confidence scores [14,31]. PFP has been shown to outperform other sequence-based methods [32-34] and has been enormously successful in international assessments of methods for function prediction (AFP-SIG '05 [35] and Critical Assessment of Techniques for Protein Structure Prediction CASP7, the function prediction [FN] category [36]). In the previous studies, we have demonstrated that PFP is superior to the other methods not only in terms of the accuracy of function assignment but also in its larger coverage for genome-scale annotation [14].

**Figure 1:**
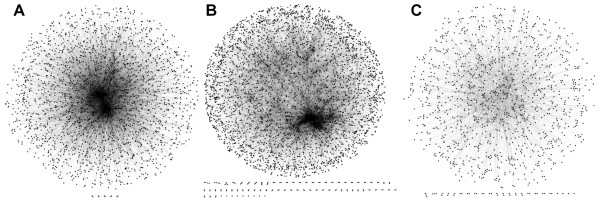
**Protein-protein interaction networks used in this work**. Networks are visualized by Cytoscape [58]: **A**, *E. coli*; **B**, *S. cerevisiae*; **C**, *P. falciparum*.

Here, we examine the utility of applying PFP predictions to genomes of three organisms, *Escherichia coli*, *Saccharomyces cerevisiae *(baker's yeast), and *Plasmodium falciparum *(malaria). The malaria genome is used as an example of a poorly annotated organism for which new annotation provides extensive interesting and useful functional knowledge. Taking advantage of PFP's larger function annotation coverage, more than 90% of proteins encoded in each genome are annotated. In order to investigate the structure of the functional space occupied by each proteome, we represented the mutual functional similarity of proteins in a form of network named the *functional similarity network*. To the best of our knowledge, this is the first of its kind introduced to investigate the structure of protein function space. Weston *et al. *proposed to consider the pairwise sequence similarity of many proteins in a database, which is named the protein similarity network, to improve the database search accuracy [37]. However, the focus of their work is to improve the database search accuracy but not the investigation of the network property of the protein similarity. Four different functional similarity networks are generated by using the function annotation in the three GO categories, namely, Biological Process (BP), Cellular Component (CC), Molecular Function (MF), and also by using the *funSim *score, which evaluates the overall functional similarity among the three GO categories. *funSim *uses the hierarchical structure of GO and information content of common ancestors of predicted and actual terms [14,38]. Analyses of the network properties of the functional similarity networks in comparison with the PPI networks (Fig. 1) revealed interesting characteristics: First, most of the functional similarity networks as well as the PPI networks are scale-free, following the power-law distribution. However, the functional similarity networks are distinct from the PPI networks by their modularity as indicated by the average clustering coefficient. Moreover, the *funSim *network distinguishes itself from the single GO-score networks by showing a higher clustering degree exponent value and thus exhibiting a higher tendency to be hierarchical, although the clustering degree exponent value seems to be sensitive to the similarity threshold value used to construct the *funSim *score network. Interestingly, the hierarchy of the biological network was first observed in metabolic pathway networks [39]. This might imply that the *funSim *score of the three organisms studied somewhat captures the structure of relationships between proteins in pathways. Additionally, we analyze functional similarity of proteins in sub networks in PPI networks and local regions of the genomes. We present several interesting and potentially useful individual cases from each of the analysis, and provide extensive supplementary data for all of the methods discussed.

## Results

### Enrichment of function annotation by PFP

We have previously shown that PFP can make more accurate function prediction than existing methods and also it can significantly increase the coverage of the function assignment to a genome [13,14]. The summary of function assignments to the three genomes used in this study, *Escherichia coli *K-12, *S. cerevisiae*, and *P. falciparum*, is shown in Table 1. PFP provides high confidence function prediction (*i.e. *prediction with the confidence score ≥ 0.8) to a significant number of genes with unknown function even to the two very well annotated genomes, *E. coli *and yeast. As for the malaria genome, which is less well annotated, the number of genes with annotation is doubled by PFP's function prediction. Consequently, more than 90% of genes in all the three genomes have function information.

**Table 1:** Number of protein genes with annotated/predicted function.

Organism	Total	Annotated ^a)^	Predicted with high confidence (≥ 0.8)	Predicted with medium confidence ≥ 0.6	Predicted with low confidence ≥ 0.4	Previously annotated and predicted with high confidence (≥ 0.8)
*E. coli *K-12	4381	3646 (83.2%)	523	696	733	4169 (95.2%)
*S. cerevisiae*	6690	5496 (82.2%)	932	1116	1187	6428 (96.1%)
*P. falciparum*	5270	2209 (41.9%)	2575	3025	3060	4784 (90.8%)

In the parentheses, the percentage of the genes relative to the total number of genes in the genome is shown.

a) The number of genes with function annotation in the GOA database.

**Figure 2:**
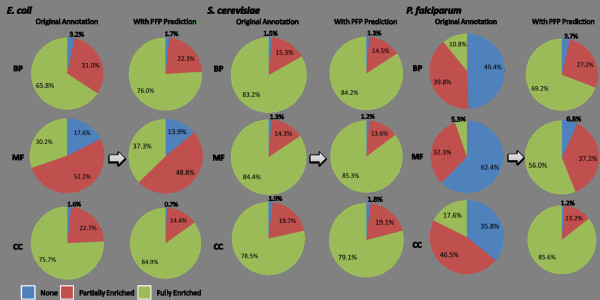
**Enrichment of function annotation in protein-protein interaction networks**. The networks show a total of 8565, 1376, and 2542 interactions for *E. coli*, *S. cerevisiae*, and *P. falciparum*, respectively. The fraction of interactions where both proteins are not annotated (none), interactions where one of the two proteins are annotated (one), and interactions where both proteins are annotated (both) are shown in the original annotation in the GOA database and after adding high confidence function prediction by PFP. Enrichment of three categories of GO, BP, MF, CC, are shown separately.

Figure 2 shows functional enrichment by PFP in the context of PPI networks of the three organisms. On a broad scale, the increase in functional knowledge for a PPI network can be described by the enrichment of annotated individual interactions. These interactions can be either (1) fully enriched, where both of the proteins involved are annotated with some functional term, (2) partially enriched, where only one of the two proteins is annotated, or (3) not have any functional terms annotated to either of the interacting partners. The increase of fully enriched interactions in *yeast *is nominal; around 1% for all the GO categories, since interactions in this organism have been already well annotated (around 80% for all the GO categories have been annotated) (Fig. 2, middle). For the cases of *E. coli*, about 10% increase of fully enriched interactions is observed in each GO category (Fig. 2, left panel). For the malaria genome, we see a significant increase in the number of fully enriched interactions in the three GO categories (Fig. 2, right panel): In the BP category, the fully enriched interactions increased from 10.8% to 69.2% (58.4 percentage point increase), while 50.7 percentage point increase is observed in the MF category. The increase is largest in the CC category (68 percentage point, from 17.6% to 85.6%). The magnitude of the increase in malaria interactions compared to *E. coli *or yeast interactions is attributed to the fact that only ~40% of the proteins encoded in the malaria genome were previously characterized, whereas upwards of 75-80% were such in both *E. coli *and yeast (Table 1).

### Functional similarity network by PFP

**Figure 3:**
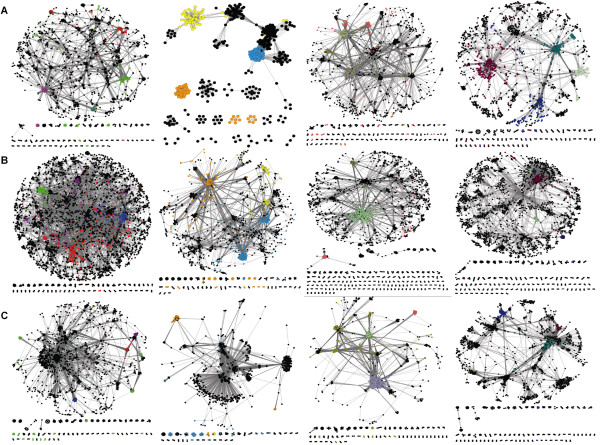
**Functional similarity networks**. A, *E. coli*; B, *S. cerevisiae*; C, *P. falciparum*. From left to right, *BP-score*, *CC-score*, *MF-score*, and *funSim *matrices. Nodes represent individual proteins and edges represent a category *GOscore *or *funSim *of ≥ 0.95. Individual clusters in the functional similarity networks are highlighted in color to show functional category of proteins. For the *BP-score *networks (left panels), green nodes represent proteins involved in transcription (GO:0006350 and its children nodes), blue nodes represent proteins involved in transport (GO:0006810), purple nodes represent proteins involved in pathogenesis (GO:0009405) (for *P. falciparum*, Fig. 3C) or signaling (GO:0007165) (for *E. coli *and yeast, Fig 3A,B), and red nodes represent proteins involved in protein modification (GO:0043687). For the *CC-score *networks (the second panels from the left in Fig. 3), yellow nodes represent proteins localized in the membrane (GO:0016020), orange nodes represent proteins localized in the ribosome (GO:0005840), and blue nodes represent proteins localized in the cell wall (GO:0005618) (for *E. coli*) or in the nucleus (GO:0005634) (for malaria and yeast). For the *MF-score *networks (the second panels from the right), light green nodes represent proteins which bind ATP (GO:0005524), pink nodes represent proteins which bind rRNA (GO:0019843), light purple nodes represent proteins which bind ions (GO:0043167), and olive nodes represent proteins exhibiting transporter activity (GO:0005215). For the *FunSim *networks (the panels on the right), burgundy nodes represent proteins which bind ATP (GO:0005524), blue nodes represent proteins localized in the ribosome (GO:0005840), and light green nodes represent proteins exhibiting transmembrane receptor activity (GO:0004888).

The previous section shows that PFP significantly enriches the function annotation in the three genomes, which facilitates analysis of the whole landscape of the functional space occupied by the genomes. In this work, we represent the functional similarity of genes in a genome as a network which is named the *functional similarity network *(Fig. 3). In the functional similarity network, nodes represent proteins with function assignment and edges between proteins denote functional similarity between them. For each genome, four networks are constructed by considering the similarity scores in the three individual GO categories, *i.e.*, *BP-score*, *CC-score*, and *MF-score *separately (Eqn. 11), and the *funSim *score (Eqn. 12). Figure 3 visualizes functional similarity networks in which protein pairs with a similarity score of 0.95 or higher are connected by edges. We also analyze networks with two different threshold values of the similarity score, 0.8 and 0.99. These networks intuitively represent overall functional space of proteins in a genome. The structure of the functional similarity networks changes as different threshold values are used. Obviously, the number of edges in a functional similarity network increases and the network becomes denser as a smaller threshold value is used for connecting edges. Table 2 shows the number of edges for the functional similarity networks using three threshold values, 0.80, 0.95, and 0.99. Here we first discuss the functional similarity networks using the threshold value of 0.95 (Fig. 3) and later analyze how the network properties change by using different threshold values. In Table 3, the parameters of the functional similarity networks for a threshold of 0.95 are underlined.

**Table 2:** Size of the functional similarity networks.

Organism	Number of Nodes ^a)^	Functional Similarity Category	Number of Nodes with 2+ Edges ^b)^/Edges		
			0.80 ^c)^	0.95	0.99
*E. coli *K-12	4169	BP	3085/208664	2169/44063	1497/13893
		CC	1603/584156	600/19862	425/17252
		MF	3033/321422	2161/74164	998/9576
		funSim	2901/121999	1172/7003	414/2648
*S. cerevisiae*	6428	BP	4622/253711	4070/72191	3270/33282
		CC	4442/3246553	2717/113947	2208/83648
		MF	4293/826942	3246/87173	1871/14257
		funSim	3879/48115	1755/10679	954/5431
*P. falciparum*	4784	BP	3968/1730159	2356/50180	1346/18444
		CC	1696/443757	1201/19154	1021/9524
		MF	4057/2619387	3788/1658678	1098/9977
		funSim	4002/208085	1521/14075	536/3134

**a) **The number of proteins which have annotated function or high confident predicted function (the last column in Table 1).

**b) **The number of proteins (nodes) which have at least two edges so that the clustering coefficient can be computed.

**c) **The threshold value of the functional similarity score to connect an edge between pairs of nodes.

**Table 3:** Network parameters of the functional similarity networks.

Parameter Type	Organism	PPI ^a)^	BP ^b)^	CC	MF	*funSim*
Degree exponent (*γ*)	*E. coli*	1.38	0.74 0.99 1.14	-0.05 0.37 0.24	0.52 0.85 1.33	0.93 1.37 1.16
	*S. cerevisiae*	1.80	0.90 1.22 1.23	0.13 0.83 0.80	0.51 0.96 1.15	1.32 1.31 1.13
	*P. falciparum*	1.60	0.35 1.02 0.89	0.09 0.73 0.72	0.25 0.21 0.93	0.94 1.27 1.22

Cluster coefficient <*C(k)*>	*E. coli*	0.08	0.74 0.75 0.77	0.67 0.85 0.79	0.82 0.74 0.69	0.65 0.49 0.45
	*S. cerevisiae*	0.10	0.50 0.63 0.58	0.75 0.77 0.77	0.72 0.72 0.62	0.46 0.46 0.50
	*P. falciparum*	0.01	0.70 0.74 0.60	0.75 0.86 0.77	0.88 0.82 0.75	0.44 0.64 0.62

Clustering degree exponent (*β*)^c)^	*E. coli*	0.75	0.31 -0.08 0.06	0.01 -0.19 -0.22	0.40 0.40 0.55	0.51 1.29 0.52
	*S. cerevisiae*	1.26	0.45 0.11 0.38	0.08 -0.05 -0.02	0.13 0.40 0.50	2.12 1.39 0.67
	*P. falciparum*	0.20	-0.20 0.51 0.42	0.26 0.57 0.39	-0.15 0.34 0.10	0.80 1.39 1.15

a) The PPI networks shown in Figure 1.

b) The degree distributions of the functional similarity networks (Fig. 3) are fit to the power-law distribution, P(k) ~ k^-*γ *^and the value of *γ *(the degree component) is computed. The values for the networks with the similarity score threshold value of 0.80 (top), 0.95 (middle, underlined), and 0.99 (bottom) are shown. Only edges with the threshold value or higher are considered.

c) The average clustering coefficient *C(k) *relative to the degree *k *is fit to the clustering-degree function, C(k) ~k-^*β*^. For the PPI, the data with k ≥ 10, while data with k ≥ 100, k ≥ 30, and k ≥ 10 are used for the functional similarity networks with the similarity score threshold value of 0.80, 0.95, and 0.99.

### The functional similarity network using the 0.95 for the similarity threshold value

**Figure 4:**
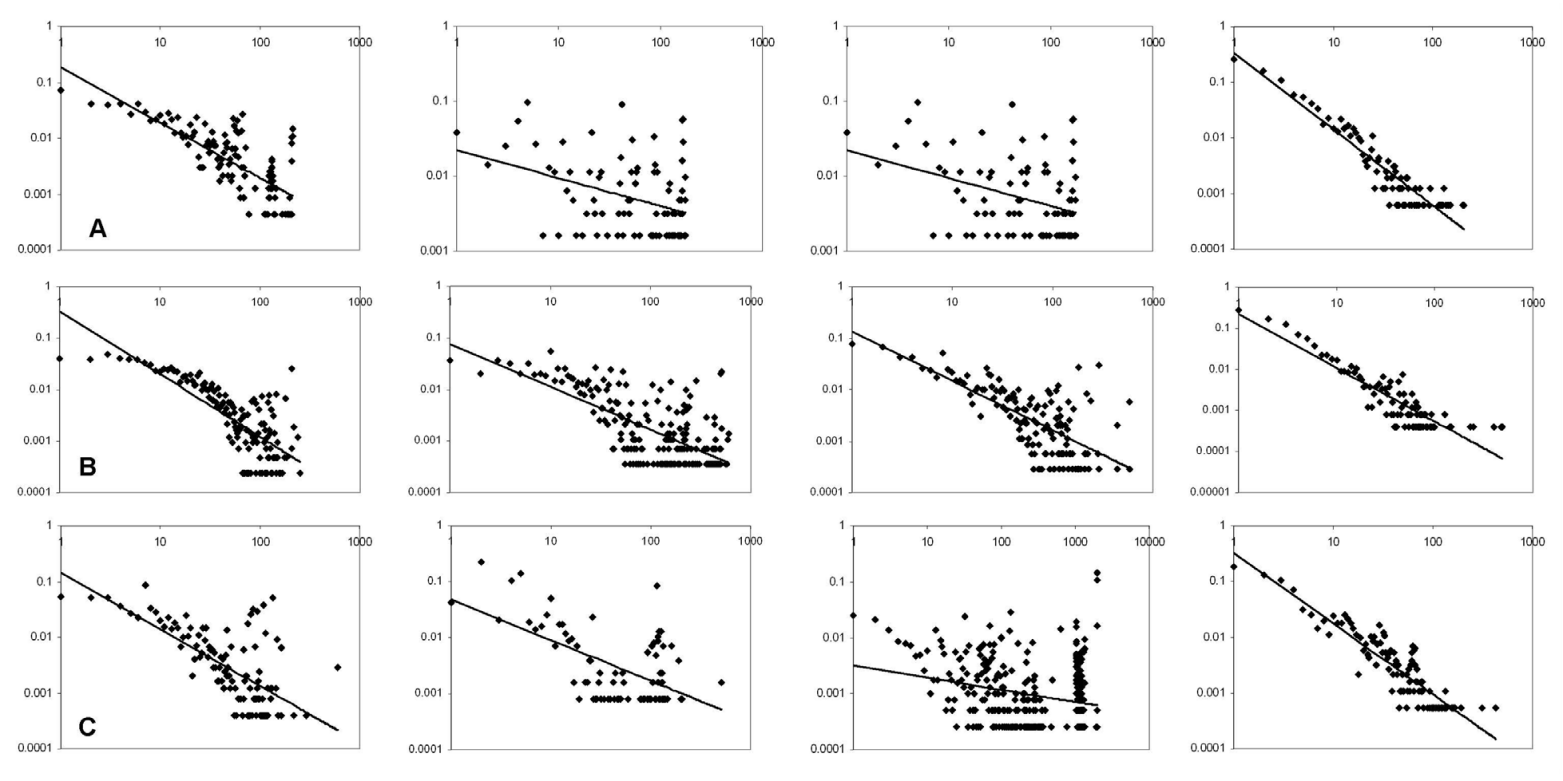
**Degree distribution of the functional similarity networks**. The similarity threshold value of 0.95 are used to connect edges. The X-axis is the number of interactions, *k *(the degree of interactions) and the Y-axis is the probability of proteins with a certain number of interactions, *P(k)*. Both axes are log scaled. The dotted line is fit to the data to compute the degree exponent, *γ*, in the power-law degree distribution: *P(k)~ k*^-*γ*^. **A**, *E. coli*; **B**, *S. cerevisiae*; **C**, *P. falciparum*. From left to right, the *BP-score*, *CC-score*, *MF-score*, and the *funSim *score. The degree exponent values are shown in Table 3. The *R*^*2 *^value of the fitted line to each distribution is as follows. *E. coli*: 0.579 (BP), 0.144 (CC), 0.472 (MF), 0.872 (funSim); *S. cerevisiae*: 0.585 (BP), 0.481 (CC), 0.505 (MF), 0.798 (funSim); *P. falciparum*: 0.466 (BP), 0.345 (CC), 0.068 (MF), 0.825 (funSim).

In all of the functional similarity networks (Fig. 3), a majority of the proteins are included in the largest connected component, which holds 74.0% (the *CC-score *network of *E. coli*) to 97.5% (The *BP-score *network of yeast) of the proteins. The degree distribution of the networks, *i.e. *the fraction of proteins, *P(k)*, with a given number of connections, *k*, (Fig. 4) shows that most of them, except for a couple of networks, follow the power-law, P(k) ~ k^-*γ *^[40]. The degree exponent *γ *ranges from 0.21 (the network for *MF-score *in *P.falciparum*) to 1.37 (the *funSim *score network in *E. coli*), which indicates that there are a small number of "hub" proteins with functional similarity to many other proteins (Table 3, top rows). The degree exponent values (*γ*) of the functional similarity networks of the *BP-*, *CC*-, and *MF-score *are smaller than those of the PPI networks shown in Figure 1, which means that the GO-score networks have larger clusters (*i.e. *hub proteins with a larger degree) and less proteins with a small degree than the PPI networks. Moreover, small *R*^*2 *^value of the *CC-score *networks (shown in the legend of Fig. 4) indicates that they do not fit well to the power-law. This is visually evident, for example, in the *CC-score *network of *E. coli *and the *MF-score *network of malaria (Fig. 3). The *funSim *networks have smaller dominant hub proteins than the *BP-*, *CC-*, and *MF-score *networks as shown by their larger *γ *values (Table 2). This is natural as a hub protein in the *funSim *network needs to have similarity in all of the *BP-*, *CC-*, and *MF-score *with neighboring proteins and thus tends to have fewer edges.

The middle rows in Table 3 show the clustering coefficients for the networks computed as per the description in methods section. We found that the PPI and the functional similarity networks are clearly distinguished by the clustering coefficient with the latter having larger modularity (*i.e. *larger values in the clustering coefficient). Single *GO-score *networks have larger modularity as compared with the *funSim *networks. The malaria *CC-score *network has the largest clustering coefficient value (0.86), which is also evident from how it looks (Fig. 3C, the second network from the left). The *funSim *networks have slightly lower modularity than the single *GO-score *networks for the same reason that they have fewer hub proteins, *i.e. *the edges need to satisfy the more severer condition of functional similarity.

**Figure 5:**
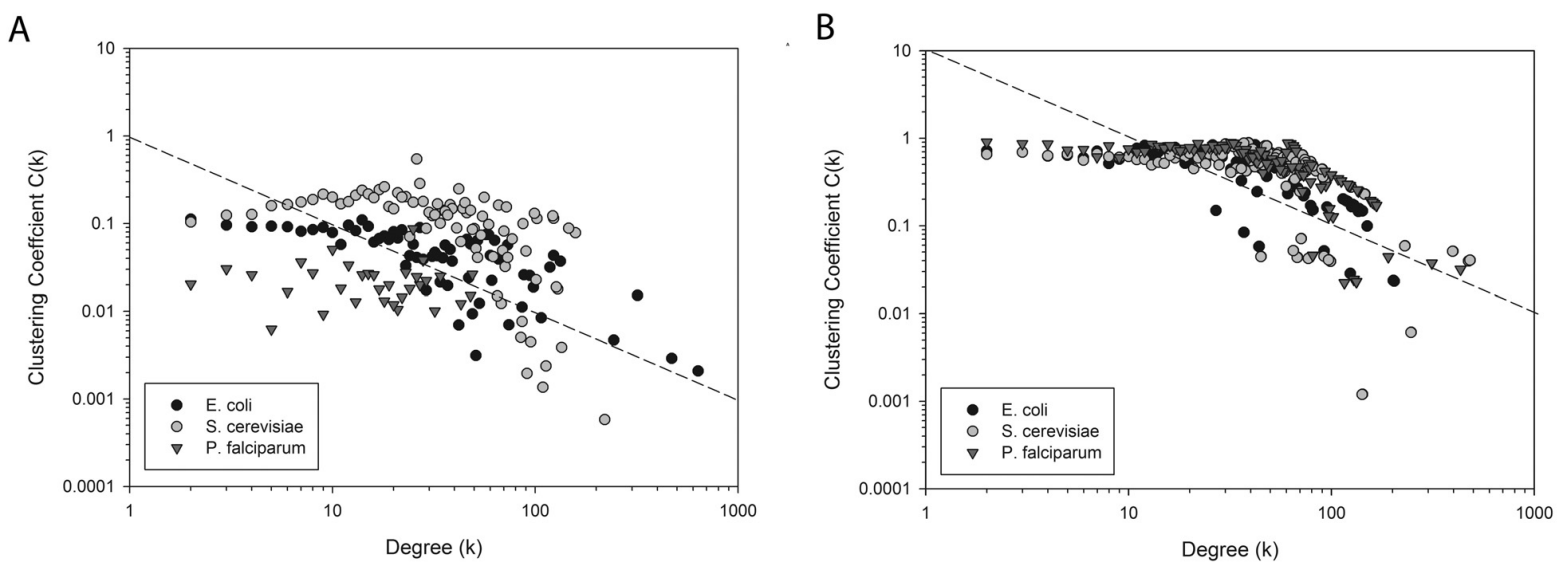
**Hierarchical modularity of networks**. *C(k) *is plotted relative to k. **A**, the PPI networks; **B**, the *funSim *networks. The dotted lines corresponds to *C(k) ~ k*^*-1*^.

We further investigated the fit of the networks to the hierarchical model proposed by Ravasz *et al. *[39]. The hierarchy in their model is quantitatively characterized by comparison of the clustering coefficient of a node with *k *links to the scaling law, *C(k) ~ k*^*-1*^. The last rows in Table 3 show the clustering degree exponent (*β; C(k) ~ k*^-^^*β*^) of each network. Consistent with previous studies [41-43], the PPI networks of *E. coli *and yeast show hierarchy, indicated by a *β *value close to 1.0 (Fig. 5A). It is rather interesting to notice that the PPI network of malaria does not show the hierarchy. The apparent dissimilar behavior of the malaria PPI network might be due to the smaller coverage of the proteins in its PPI network. Compared to the *E. coli *and yeast PPI networks which include more than 55% of total known proteins, the malaria PPI network covers only 23.1% of its proteins. The individual *GO-score *networks show less dependency of the *C(k) *value to the degree *k *and thus do not exhibit hierarchy as shown by their small clustering degree exponent values. However, the clustering coefficient of the *funSim *network is well approximated by *C(k) ~ k*^*-1 *^(Fig. 5B). It is an interesting observation that hierarchy of the network arises for the *funSim *score that integrates single GO-scores, which do not show hierarchy individually. It might imply that the *funSim *score somewhat captures properties of the metabolic networks. Although both the PPI and the *funSim *network show hierarchy, they are different in the range of the clustering coefficient values (*i.e. *the y-value in Fig. 5), with the latter having larger values.

### Effect of changing the similarity threshold value for connecting edges

In addition to the networks with the similarity threshold value of 0.95 which are discussed above, we examine the networks using a smaller threshold value, 0.80, and a higher threshold value, 0.99 to understand how the network structure changes. The total number of edges significantly increases by using a more permissive threshold value (0.80) for connecting edges and decreases with a larger threshold value (0.99) (Table 2). As the networks become denser with more edges (using the threshold value of 0.80), the number of highly connected nodes increases, which reflects to the decrease in the degree component (*γ*). This trend is evident especially for the CC-score networks, for which the degree component values are too small for them to be power-law networks. The *funSim *networks of the three organisms constantly have high degree exponent values. The average clustering coefficient values (middle rows in Table 3) are relatively less affected by the change of the similarity threshold values for drawing edges. Thus all networks with all three similarity threshold values examined are modular.

**Figure 6:**
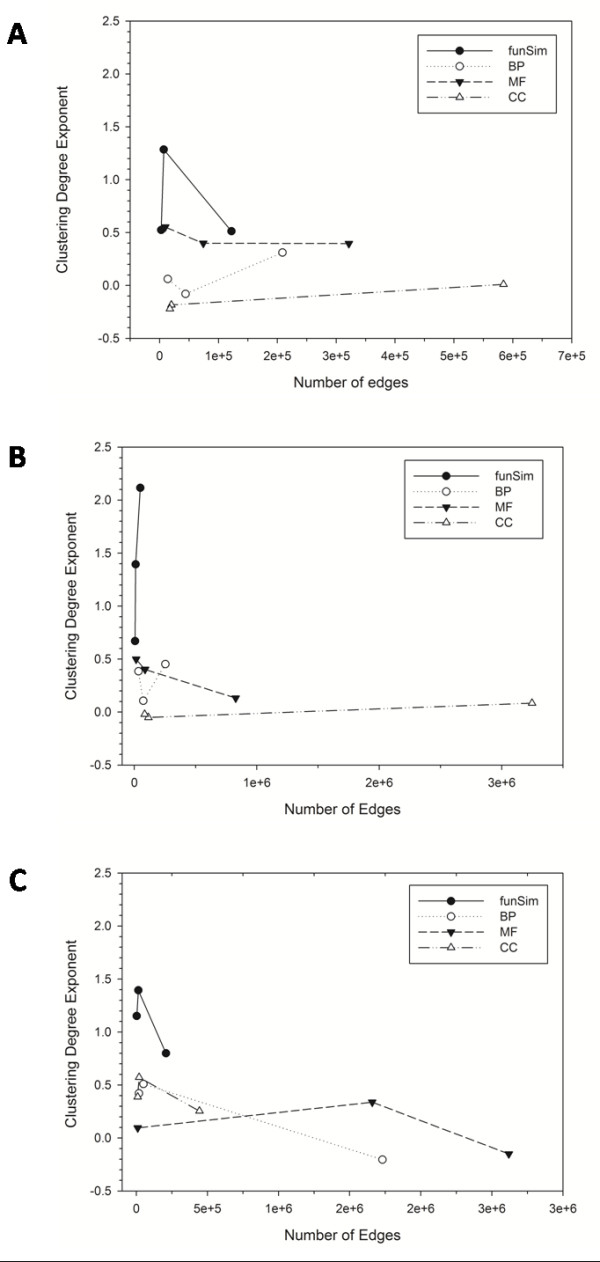
**The clustering degree exponent value of the functional similarity networks relative to the number of edges in the networks**. **A**, *E. coli*; **B**, *S. cerevisiae*; **C**, *P. falciparum*.

Looking at the clustering degree exponent values, *β*, (the last rows in Table 3), none of the single-GO score networks exhibits significant hierarchy (*i.e. *the *β *value of around 1.0) by changing the similarity threshold value. The *funSim *networks of malaria consistently show hierarchy for all the similarity threshold values. The *β *value for the *E. coli funSim *networks drops to around 0.5 by changing the similarity threshold value from 0.95 to a smaller (0.80) and also to a larger (0.99) value. In the case of yeast *funSim *network, lowering the similarity threshold value still keeps a high *β *value but raising the threshold value to 0.99 drops it to 0.67. Thus, referring to the originally proposed scaling law for the hierarchical network [39], which has the *β *value of 1.0, *E. coli funSim *networks with the similarity threshold value of 0.80 and 0.90 as well as the yeast *funSim *network with the similarity threshold value of 0.99 may not be fully qualified as hierarchical. However, as Figure 6 and Table 3 show, the *funSim *networks have a higher *β *value, and thus tend to be more hierarchical than the single *GO-score *networks.

To summarize, both PPI and most of the newly described functional similarity networks are scale-free. The PPI network and functional similarity networks (namely, *funSim*, *BP-score*, *CC-score*, and *MF-score *networks) are distinguished by their modularity, with the latter networks showing significant modularity with high clustering coefficient values while the PPI does not. Lastly, the *funSim *network is different from the single GO-score networks by exhibiting a higher tendency to be hierarchical (*i.e. *showing a higher *β *value). However, note that the *β *value of *funSim *networks seem to be sensitive to the similarity threshold value and E. coli and yeast *funSim *networks drop their *β *value to less than 1.0 when the similarity threshold value is changed to 0.80 and 0.99.

### Annotating PPI subnetworks

Next, we examine subnetworks in the PPI networks to see how many edges in them are present in the functional similarity networks. Here, the functional similarity networks with the similarity threshold value of 0.95 are used. We compare the number of edges in subnetworks connecting nodes with common annotations assigned in the GOA database and edges with nodes with common predictions by PFP (Table 4). Edges with common annotations for both nodes did not increase much in *E. coli *and yeast by PFP's prediction. Particularly, there is no increase in the number of edges with common annotations in yeast. This is because yeast PPI networks have been already well annotated as shown in Figure 2. In contrast, 131 out of 155 subnetworks of malaria are enriched by the PFP prediction. The number of edges with common annotations increased four times (from 241 to 972 edges) in malaria.

**Table 4:** Enrichment of function annotation to subnetworks.

Organism	# of subnetworks	# of edges	# of edges with common annotation ^a)^	# of edges with common annotation or prediction ^b)^	# of edges with common predicted annotation ^c)^	# of subnetworks with functionally enriched edges ^d)^
*E. coli*	632	6401	2689 (42.0%)	2718 (42.5%)	29 (0.5%)	17
*S. cerevisiae*	1148	38108	29407 (77.2%)	29407 (77.2%)	0 (0%)	0
*P. falciparum*	155	2578	241 (9.3%)	972 (37.7%)	731 (28.4%)	131

a) Edges connecting two nodes with (at least one) common GO annotation in the GOA database. In the parentheses, the fraction of the edges relative to the total number of edges is shown.

b) Edges connecting two nodes with (at least one) common annotation in the GOA database or common GO term prediction by PFP.

c) Edges connecting two nodes with common GO term prediction by PFP.

d) Subnetworks where the number of edges with common annotation increased by considering PFP prediction.

Since malaria has the largest annotation enrichment among the three organisms (Fig. 2, right panel and Table 4), below we focus on annotations given to the malaria PPI network. Following a previous work [44], we examine annotation given to subnetworks of the PPI. A subnetwork is identified as all proteins connected to a common centroid protein and the edges among them. The statistical significance of the number of edges in a subnetwork is tested by computing the connectivity coefficient (Eqn. 5) compared with 100 randomized networks. Those subnetworks with a p-value of below 0.05 by the t-test (Eqn. 6) are identified as targets for discussion. We identified 155 subnetworks which hold 716 (97.3%) of the proteins in the entire PPI network.

**Figure 7:**
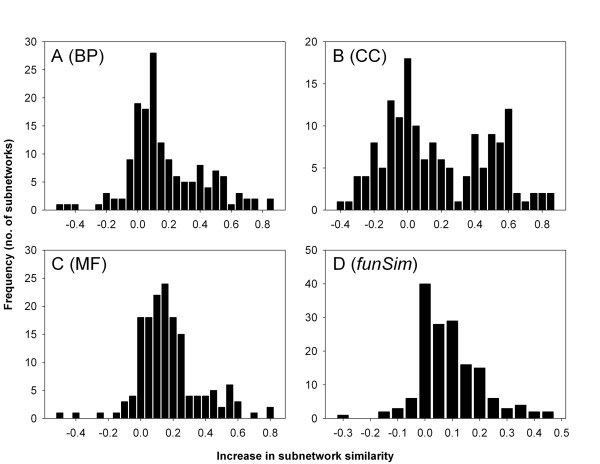
**The increase of the average score similarity of subnetworks of *P. falciparum***. The score before and after adding function prediction by PFP to the 152 subnetworks are compared. **A**, *BP-score*; **B**, *CC-score*; **C**, *MF-score*; and **D**, *funSim *score.

Each target subnetwork is tested for overrepresentation of GO terms using only previously known annotations and then using known *and *predicted terms by PFP. The false discovery rate (FDR) correction of the hypergeometric distribution (Eqn. 15) is used to evaluate the statistical significance of overrepresented GO terms in a subnetwork. For malaria, we found six subnetworks in which no functional terms were overrepresented in the original annotation in the GOA database. To these we assigned 422 new annotations. In 146 other subnetworks we were able to identify a total of 6,391 new overrepresented GO terms, with an average annotation gain of 591%. To evaluate the consistency of newly predicted annotations with previously known annotations, we used the *funSim *score (Eqn. 12) to compare all of the terms within each subnetwork. It is a general assumption that interacting proteins are involved in the same or coordinating biological pathways and coexist in the same locations within the cell [45,46]. For malaria, newly predicted functional terms had a positive effect on the majority of the subnetworks as shown in the histograms (Fig. 7). On an average, *BP-score*, *CC-score*, *MF-score*, and *funSim *score similarity increased by 0.198, 0.189, 0.195, and 0.108, respectively. Thus, not only does the addition of predicted terms effect in an increase in the functional information available for annotating a subnetwork, but it also tends to refine the overall annotation for that subnetwork.

**Figure 8:**
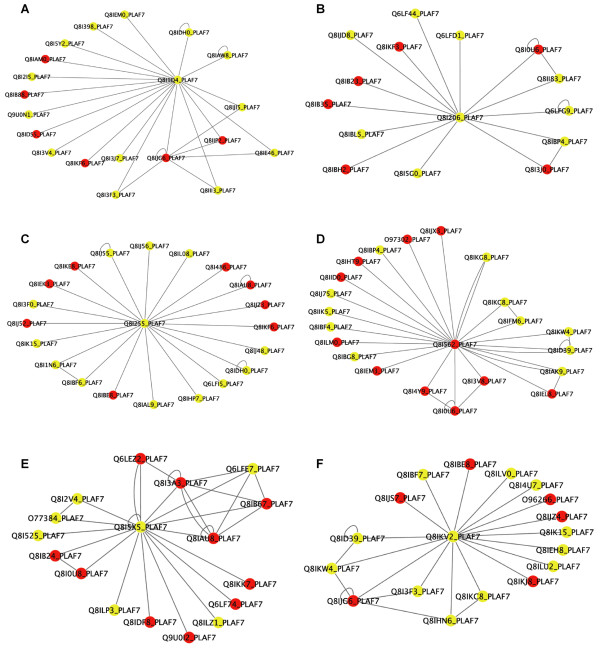
**Protein-protein interaction subnetworks described in Table 3**. Proteins in the center of the subnetworks are: **A**, Q8I1Q4; **B**, Q8I206; **C**, Q8I255; **D**, Q8I562; **E**, Q8I5X5; **F**, Q8IKV2. Previously annotated proteins are colored red and proteins with functions predicted by PFP are colored yellow. Circular edges are self-interactions detected for the proteins. See Table 5 for function annotations of the proteins in these subnetworks.

Below we present six individual cases of interesting new annotations to subnetworks in *P. falciparum*. The previous and new annotations for each of these examples are provided in Table 5, and visual representations of the analyzed subnetworks are provided in Figure 8. The subnetwork centered by the protein Q8I1Q4 (Fig. 8A) contains 20 proteins, 14 of which are newly annotated by PFP prediction. Among the six previously annotated proteins, representative functionality by our analysis deals with chromosome/chromatin packing. When new high-confidence predicted annotations are considered, some new functional terms arise as statistically overrepresented in the subnetwork. These include several functions relating to the cytoskeleton (actin binding and myosin, cytoskeleton-dependent transport) and nuclear-directed transport. The theme of these new annotations tends to suggest that interactions among this group of proteins may reveal a transport mechanism, potentially for moving proteins involved in chromosome packaging into the nucleus. Next, protein Q8I206 centers a subnetwork of proteins which we were unable to characterize using only known annotations (Fig. 8B). Nine of the 15 proteins are annotated with high-confidence predictions, revealing two related groups of functional terms as potential representatives of the overall function of the subnetwork. The first of these groups is related to nucleic acid binding and transport and localization, and the second is related to carbohydrate metabolism.

**Table 5:** Annotations of highly interconnected PPI subnetworks in malaria.

Centroid	Proteins (Edges)	Previous annotations (GO)	P-value ^a)^	New annotations with PFP (GO)	P-value
Q8I1Q4	20 (24)	chromatin assembly or disassembly (0006333)	0.043	Myosin I binding (0017024)	0.004
		chromosome organization and biogenesis sensu Eukaryota (0007001)	0.048	Cytoskeleton-dependent intracellular transport (0030705)	0.028
				Structural constituent of nuclear pore (0017056)	0.020
				mitotic cell cycle (0000278)	0.040
				Nuclear export (0051168)	0.031
				Nuclear import (0051170)	0.043
					
Q8I206	15 (16)	---	---	Nucleic acid transport (0050567)	0.003
				Nucleobase, nucleoside, nucleotide and nucleic acid transport (0015931)	0.004
				Transport (0006810)	0.045
				Localization (0051179)	0.026
				Regulation of gluconeogenesis (0006111)	0.017
				Glucosyltransferase activity (0046527)	0.032
					
Q8I255	21 (21)	Signal transducer activity (0004871)	0.002	Hydrolase activity (0016787)	0.023
		Receptor binding (0005102)	0.014	Translation regulator activity (0045182)	0.003
		Pathogenesis (0009405)	0.009	Autotransporter activity (0015474)	0.041
		Extracellular region (0005576)	0.022	Structural constituent of nuclear pore (0017056)	0.023
				Localization (0051179)	0.008
				Negative regulation of lymphocyte activation (0051250)	0.026
				Peroxisome degradation (0030242)	0.023
				Microtubule cytoskeleton organization and biogenesis (0000226)	0.008
				Protein catabolism (0030163)	0.046
				Intermediate filament cytoskeleton (0045111)	0.040
					
Q8I562	25 (31)	Cellular protein metabolism (0044267)	0.028	Cell death (0008219)	0.030
		Protein folding (0006457)	0.022	RNA localization (0006403)	0.006
				Anterior/posterior axis specification (0009948)	0.025
				Anterior/posterior pattern formation (0009952)	0.025
				Cytoskeleton organization and biogenesis (0007010)	0.027
				Myosin II (0016460)	0.010
				Actin cytoskeleton (0015629)	0.012
					
Q8I5X5	18 (27)	Transferase activity (0016740)	0.017	ATP binding (0005524)	0.0001
		Glycolysis (0006096)	0.003	Cellular protein metabolism (0044267)	0.020
		Macromolecule catabolism (0009057)	0.002	Catalytic activity (0003824)	0.047
		Kinase activity (0016301)	0.036	Intermediate filament cytoskeleton (0045111)	0.030
				Cytoskeleton-dependent intracellular transport (0030705)	0.002
					
Q8IKV2	18 (22)	Chromatin binding (0003682)	0.011	Adenyl nucleotide binding (0030554)	0.019
		Chromatin assembly or disassembly (0006333)	0.043	Transcription coactivator activity (0003713)	0.007
		Chromosome organization and biogenesis sensu Eukaryota (0007001)	0.048	RNA-mediated posttranscriptional gene silencing (0035194)	0.018
				Translation regulator activity (0045182)	0.008

a) The P-value is computed by Eqn. 15.

The next two examples are potentially more interesting, especially with regard to the known pathogenicity of the Malaria plasmodium. The group of 21 proteins centered on Q8I255 was previously annotated with terms directly related to pathogenesis (pathogenesis, extracellular, signal transduction) (Fig. 8C). After providing predicted annotations for 13 of those 21 proteins, several other functions that may be related to particular pathogenic mechanisms were revealed. Particularly interesting are the terms "translation regulator activity", "negative regulation of lymphocyte activation", "microtubule cytoskeleton organization and biogenesis", and "peroxisome degradation". Although the proteins in this subnetwork could already be associated with pathogenesis, new predicted annotations for uncharacterized proteins add direction for designing experiments to test for specific mechanisms that may be *responsible *for the pathogenic behavior. The interaction subnetwork around Malaria protein Q8I562 (Fig. 8D) also has some potential interest in the molecular mechanisms that contribute to apoptosis. Again, over half of the included proteins (14 of 25) were initially uncharacterized but could be assigned high confidence PFP predictions. Before taking new predictions into account, the cluster was annotated as being related to "cellular protein metabolism" and "protein folding". Several more interesting and specific functional terms were brought to light after including predictions. These terms are related to the cytoskeleton and protein/RNA transport and localization. Specifically, the terms "anterior/posterior pattern formation", "RNA localization", and "cell death" are closely related and signify that the protein interactions in this subnetwork are likely to be involved in the programmed re-organization of the cell leading to death, or apoptosis.

### Identifying clusters of functionally related protein-coding genes in genomes

**Figure 9:**
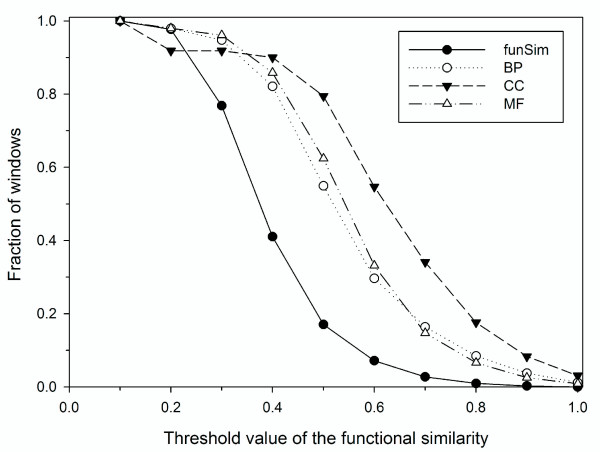
**The accumulated fraction of genomic windows in *E. coli *that satisfy the similarity threshold values**. Results for the *funSim *score and individual *GO scores *are shown.

**Figure 10:**
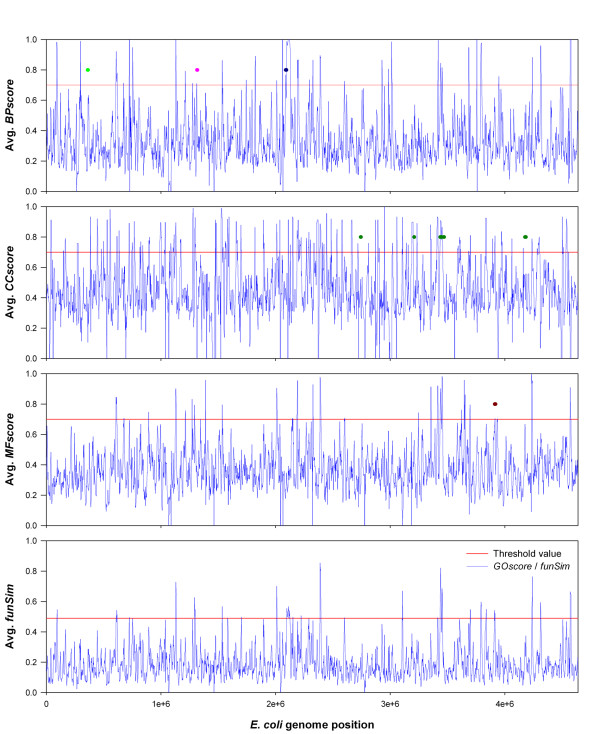
**Variability of functional similarity in the *E. coli *genome**. Functional similarity (Y-axis) here is an all-by-all category *GO score *or *funSim *average among the genes included in the local window. The X-axis is the genome position of the left-hand side of the window. The red line indicates the threshold value of functional similarity we used for individual analysis of a genome window for overrepresentation of GO terms (0.7 for each category *GO score *average, 0.49 for *funSim *average). The dots denote known clusters of functionally similar genes. For the BP graph, neon green is the *lac *operon, pink is the *trp *operon, and dark blue is the *his *operon. For the MF graph, dark red dots are ATP synthase components (atpX). And for the CC graph, dark green dots are proteins of the ribosome. The same plots for yeast and malaria genomes are not provided since they have much larger genomes (yeast and malaria have 16 and 14 chromosomes, respectively) but all the data are available on our website.

Genome proximity is known to be related to conservation of protein function, most notably in the cases of coordinately regulated groups of protein-coding genes in operons or regulons [47] and among some membrane transport proteins [48]. Similarity of phylogenetic profiles [25] and stability of local genome organization between species [49] have also revealed functional conservation among groups of genes. Here we scanned the three genomes using a window of a certain size (10 kb for *E. coli *and 30 kb for yeast and malaria genome) to identify groups of neighboring genes with significant function similarity. Windows of genes that have an overall categorical similarity (one or more of *MF-score*, *BP-score*, or *CC-score*) of greater than 0.7 or a comprehensive similarity (*funSim*) of greater than 0.49, including new functional terms predicted by PFP, were considered for analysis. The threshold values, 0.7 and 0.49, are chosen to roughly match the number of windows to be selected with the number of known regulons in *E. coli*. According to the RegulonDB database (June 2009 release) [47], there are 374 regulons in the *E.coli *genome. Using 0.7 in MF, BP, and CC score selects 339 (14.7%), 377 (16.4%), and 779 (34.1%), respectively, and 0.49 in the *funSim *score selects 437 (18.8%) windows (Fig. 9). For example, the windows with regulons of ribosomal subunits (rplQ, rpoA, rpsJ, etc.), flagellar proteins (two windows: flgA, flgB, etc. and fliE, fliF, etc.), his operon (hisL, hisG, etc.), and psp operon (pspF, pspA, etc.), satisfy these threshold values. Figure 10 illustrates the functional similarity scores along the *E. coli *genome. Some of the known operons are marked in color.

As with the PPI subnetworks, each genome window identified as a target was tested for overrepresentation of GO functional terms using only previously known annotations and then using known *and *predicted terms by PFP using the hypergeometric distribution (Eqn. 15). The percent difference between these two scenarios is used as our standard measure of annotation gain. The summary of the increase of the annotation to the genome windows is shown in Table 6. Again, as was the case with annotation of PPI subnetworks, we would expect to find that applying predicted terms to groups of proteins in *E. coli *and yeast would yield some, but not an extensive, degree of annotation gain. This indeed turned out to be true. For *E. coli *and yeast, we were able to annotate 38 and 29 previously unannotated windows, respectively, in each genome. The average annotation gain computed for previously annotated windows are 49% and 14% for *E. coli *and yeast, respectively. Analysis of annotation gain among windows in the malaria genome again yielded significantly higher increases. 37% (2418 out of 6539) of windows with no previously known functional annotation were assigned with predicted GO terms by PFP. The remaining 2,735 windows for which some annotation already existed, we observed an average annotation gain of 289% (Table 7).

**Table 6:** Summary of increase in annotation in genomic windows.

Organism	Total # of windows ^a)^	Prior un-annotated windows ^b)^	Prior un-annotated windows which are annotated by PFP	Total # of GO terms added by PFP to prior un-annotated windows ^c)^	# of prior annotated windows ^d)^	# of prior annotated windows to which more GO terms are predicted by PFP	# of GO terms added to the prior annotated windows
*E. coli*	27,840	4,436	38	142	23,404	917	1750
*S. cerevisiae*	48,260	4,807	29	111	43,453	670	925
*P. falciparum*	45,036	6,539	2418	17435	38,497	2735	17286

a) These numbers include windows with genes on the plus strand, those with genes on the minus strand, and those with genes from the both strands.

b) The number of windows which include only unannotated genes in the GOA database.

c) Only overrepresented GO terms are considered.

d) The number of windows which all the included genes are unannotated in the GOA database.

**Table 7:** Examples of windows with newly annotated highly similar genes.

Organism	Position	Direction ^a)^	# of Proteins (Average *FunSim*)	New annotations (GO)	P-value ^b)^
*E. coli*	1212000 (bp)	Both	11 (0.792)	Regulation of biological process (0050789)	0.00006
				Intracellular membrane-bound organelle (0043231)	0.0000005
				Membrane-bound organelle (0043227)	0.0000005
				Intracellular organelle (0043229)	0.000004
	3016000	both	7 (0.711)	Transport (0006810)	0.002
				Establishment of localization (0051234)	0.002
yeast	chr07 798000 (bp)	minus	2 (0.793)	rRNA processing (0006364)	0.001
	chr02 165000	both	7 (0.704)	Organelle lumen (0043233)	0.001
				Membrane-enclosed lumen (0031974)	0.001
	chr14 141000	Plus	3 (0.749)	Chromatin silencing at telomere (0006348)	0.0004
				Telomeric heterochromatin formation (0031509)	0.0004
	chr15 342000	both	6 (0.760)	Signal transducer activity (0004871)	0.001
				Transmembrane receptor activity (0004888)	0.00007
				Receptor activity (0004872)	0.0002
malaria	chr03 906000	both	6 (0.722)	Phosphotransferase activity, alcohol group as acceptor (0016773)	0.001
				Transferase activity, transferring phosphorus-containing groups (0016772)	0.002
	chr06 6000	both	5 (0.891)	NADPH regeneration (0006740)	0.0001
				NADPH metabolism (0006739)	0.0001
				Nicotinamide metabolism (0006769)	0.0001
				Pyridine nucleotide metabolism (0019362)	0.0002
				Oxidoreduction coenzyme metabolism (0006733)	0.0004
				Water-soluble vitamin metabolism (0006767)	0.0004
	chr07 1296000	both	7 (0.866)	Dopamine receptor activity (0004952)	0.0001
				Amine receptor activity ( 0008227)	0.0001
				Neurotransmitter receptor activity (0030594)	0.0001
				Dopamine binding (0035240)	0.0001
				Rhodopsin-like receptor activity (0001584)	0.001
				Receptor activity (0004872)	0.001
				Neurotransmitter binding (0042165)	0.0001
				G-protein coupled receptor activity (0004930)	0.001
	chr09 144000	plus	3 (0.881)	RNA localization (0006403)	0.0002
	chr10 492000	minus	4 (0.860)	Mitotic cell cycle (0000278)	0.0001
				Negative regulation of transcription by carbon catabolites (0045013)	0.0004
				Regulation of transcription by carbon catabolites (0045990)	0.0004
				Response to nutrients (0007584)	0.0004
				Regulation of transcription by glucose (0046105)	0.0004
				Intracellular transport (0046907)	0.0003
				Establishment of localization in cell (0051649)	0.0003
	chr14 3174000	both	6 (0.800)	Autophagic vacuole fusion (0000046)	0.00001
				Organelle fusion (0048284)	0.00009
				Macroautophagy (0016236)	0.00002
				Autophagy (0006914)	0.00002

a) The direction of the DNA strands on which the genes are located.

b) The P-value of the overrepresentation of the GO term in the genes in the window

Here, we also present several individual cases of new annotation to regions in the genomes of each of the three organisms. A summary of the new annotation is shown in Table 7. The 30 kb region of malaria chromosome 3 starting at position 906,000 contains six proteins with an average GO biological process similarity of 0.722. The 30 kb region of malaria chromosome 3 starting at position 906,000 contains six proteins with an average GO biological process similarity of 0.722. After annotating four of the five previously uncharacterized proteins coded here with high confidence predictions, we found that the proteins may share involvement in phosphorylation or dephosphorylation ("phosphotransferase activity" and "transferase activity, transferring phosphorous-containing groups"). This may indicate that these neighboring proteins are involved in a common signaling or metabolic pathway. Similarly, the region of malaria chromosome 7 starting at position 1,296,000 (five proteins, average biological process similarity of 0.891) was assigned several receptor-like activities. The overrepresented terms related to several types of receptor activity give a strong indication that this region contains proteins that form complex or interact closely as part of a membrane signaling receptor. Membrane receptors and complexes of membrane proteins are well characterized as sharing genome proximity [48]. The four proteins between positions 492,000 and 522,000 of the minus strand of Malaria chromosome 10 (average biological process similarity of 0.860) were assigned several functional terms that all relate to the intrinsic cellular response to nutrients. The terms "intracellular transport", "response to nutrients", "negative regulation of transcription by carbon catabolites", and "mitotic cell cycle" could all indicate a common process involving metabolism and cellular signaling response to the presence of nutrients under particular conditions, perhaps akin to the well known *lac *operon in *E. coli*.

*E. coli *is one of the most well-characterized model organisms in terms of coordinately regulated expression in the form of operons and regulons [47]. As such, we would not expect to find many regions of the genome that could represent new examples of these molecular phenomena relating to specific pathways. However, we did find several examples including the following two where annotation of previously uncharacterized regions might indicate common involvement in processes. First, the 11 proteins within 10 kb of position 1,212,000 (average biological process similarity of 0.792) share broad annotations of "regulation of biological process" and "intracellular membrane-bound organelle". Second, the seven proteins within 10 kb of position 3,016,000 (average biological process similarity of 0.711) share similarly broad annotations of "transport" and "localization". In either case, these annotations might indicate involvement in a common complex or process in a particular membrane-bound organelle or localization pathway, and might be enough to warrant further investigation into the biological reason for the shared function.

Yeast is similarly well characterized, but we again found some examples of genomic windows where application of new high confidence predictions revealed a shared function or related functions. There are two particularly interesting examples. First, the 15 kb region of the plus strand of Yeast chromosome 14 starting at position 141,000 contains three proteins (average cellular component similarity of 0.749) that share the annotations "chromatin silencing at telomere" and "telomeric heterochromatin formation". Second, the six proteins located in 15 kb region of chromosome 15 starting at position 342,000 (average cellular component similarity of 0.760) share the related functions of "signal transducer activity" and "transmembrane receptor activity".

Details of individual protein interaction subnetworks and genomic windows, and previous and new annotations for each subnetwork and window can be found in the supplementary data.

## Discussion

In this analysis, we enriched functional annotation to the three genomes by PFP's high confidence predictions and represented the functional space occupied by the proteomes in the *functional similarity network*, where edges between proteins (nodes) denote significant functional similarity between them. To the best of our knowledge, this is the first time that structure of functional space is analyzed as a network. Taking advantage of the PFP's large annotation coverage [14], more than 90% of proteins in each genome are included in the functional similarity network (Table 1). This is a significant enrichment especially for the malaria genome, as previously only 41.9% of proteins were annotated. We defined the functional similarity of proteins using their annotated GO terms rather than other possible functional similarity metrics, *e.g. *the conventional sequence similarity, because GO terms can compare proteins in different aspects of functions (*i.e*. in different GO categories and their combinations), which may be more relevant to protein activity in the cell. Moreover, proteins with a high sequence similarity shows significant similarity in the annotated GO terms as well in majority of the cases, so protein sharing GO term similarity can be considered a superset of those sharing sequence similarity [38,50].

Our study revealed interesting characteristics of the functional similarity networks of the three organisms contrasted with the PPI networks. We analyzed the global topology of the functional similarity network by computing the degree exponent, the clustering coefficient, and the clustering degree exponent of the networks (Table 3). In general, both functional similarity networks and PPI networks follow the power-law, but they are distinct in the former showing the network modularity but the latter does not. Among the four functional similarity networks constructed by considering individual GO-scores and the *funSim *score, the *funSim *score network is different from the others by exhibiting a higher tendency to be hierarchical (*i.e. *higher clustering degree exponent value) similar to the metabolic pathway networks. However, the clustering degree exponent value seems to be sensitive to the similarity threshold value used to construct the networks and the *E. coli *and yeast *funSim *score networks drop its value below 1.0 when some similarity threshold values are used.

Unlike the current PPI network data, which provide a static view of protein interactions, the functional similarity networks change their topology as the similarity threshold value is changed. Functional similarity networks of a different similarity threshold value represent different levels of granularity of the gene function space in a genome. Investigation of the global and local structure properties of dynamically changing functional similarity networks is left as an important future work.

It is reminded that the currently available PPI networks have several limitations; they are usually incomplete and potentially include false positive and false negative interactions [51,52]. However, we expect that such limitations will not affect to this work too much since the focus of this work is the construction of the functional similarity networks and the functional enrichment by PFP. We analyzed the PPI networks as to contrast to the newly introduced functional similarity networks. As a future work, it may be interesting to compare the network properties of the functional similarity networks with other types of biological networks, such as gene regulatory networks [53,54] or gene functional networks constructed by considering different types of experimental information [55].

Individual annotation to subnetworks in the PPI networks and genome local windows identified numerous interesting cases where proteins in the subset show high coherence with other members. These results provide examples of how computational prediction can be utilized in interpreting or building hypotheses on the proteins sharing such functional association. Interestingly, there are several cases where proteins in a genome window are functionally coherent with PFP's assignment of broader, less-specific functional terms. These may not be regulons or operons, where functional roles of component genes are usually better defined. Rather, these local windows of genes may imply existence of a new type of gene clusters where genes are inter-related by much broader, higher-level functional category.

Together with the introduction of the functional similarity networks and functional coherence of individual subsets of genes, we have demonstrated the usefulness of computational function prediction by PFP. The same methods can be applied to any biologically related group of proteins. High-throughput technologies such as microarrays and mass spectrometry that identify clusters of proteins linked by common expression patterns or conditions produce datasets that would also be relevant for such an application. In the end, as PFP is a sequence similarity-based prediction method, utilizing its high confidence predictions takes a minimal time and energy commitment (~1 day to run all uncharacterized proteins for *P. falciparum*) and can have a significant impact on a researcher's ability to interpret the complex datasets that have now become the norm.

## Conclusion

We assigned function to previously uncharacterized protein genes in *Escherichia coli *K-12, *Saccaromyces cerevisiae*, and *Plasmodium falciparum *with high-confidence function prediction by the PFP method. Using the enriched function annotation, we introduced the functional similarity network which provides an intuitive representation of the functional space of a proteome. Comparison with the PPI networks revealed distinct features of the functional similarity networks. In addition, PFP's function assignment identified functionally coherent subnetworks in the PPI and local regions in the genomes. All together, this work demonstrated usefulness of the computational functional predictions by PFP.

## Methods

### Data sources

The genome sequence and annotation data for *Escherichia coli *K-12, *Saccaromyces cerevisiae*, and *Plasmodium falciparum *were obtained from the website of the European Bioinformatics Institute (EBI). Annotations qualified as "previously known" were extracted from EBI's GOA proteome datasets http://www.ebi.ac.uk/GOA/. PPI data for *E. coli *was obtained from Arifuzzaman *et al. *[5], for *S. cerevisiae *was obtained from MIPS [56], and for *P. falciparum *was obtained from the paper by LaCount *et al. *[44]. Genome position data was obtained from the website of the National Center for Biotechnology Information (NCBI) ftp://ftp.ncbi.nih.gov/genomes/.

### Computing Clustering Coefficient

The clustering coefficient of a node indicates how well the neighboring nodes to the central node are interconnected and it is used to measure the *modularity *of a network [39,40]. Concretely, it is computed as follows for a given node:(1)

where *k *is the number of neighboring nodes connected to the central node and *n *is the number of pairs of the neighboring nodes that are directly connected. To quantify the modularity of an entire network, the average clustering coefficient is computed [39,40].

### Function Prediction by PFP

GO functional terms were predicted for each sequence without any previously assigned GO terms from *E. coli*, *S. cerevisiae*, and *P. falciparum *using PFP under its optimal parameter settings, which are described below. Refer to the previous work [14] for detailed analyses of the effect of using different parameter values. Only terms predicted with high confidence (≥ 0.8) were assigned to each query sequence. The detailed description of the algorithm as well as thorough benchmark results of PFP have been reported in the previous papers [13,14]. Here we will briefly overview the PFP algorithm for readers' convenience.

The PFP algorithm predicts GO function annotations in three categories, *i.e. *MF, BP, and CC, with a statistical significance score (p-value) and the expected accuracy. For each sequence hit retrieved by a PSI-BLAST search [32], associated GO terms are scored according to the E-value provided by PSI-BLAST. Then the scores of a GO term are summed up over all the sequence hits considered. This scoring system ranks GO terms by considering both (1) their frequency of association to sequence hits and (2) the degree of similarity those sequences share with the query. A GO term, *f*_*a*_, is scored as follows:(2)(3)

where *s*(*f*_*a*_) is the final score assigned to the GO term, *f*_*a*_, *N *is the number of the similar sequences retrieved by PSI-BLAST, *Nfunc*(*i*) is the number of GO terms assigned to sequence *i*, *E_value*(*i*) is the E-value given to the sequence *i*, and *f*_*j *_is a GO term assigned to the sequence *i*. *P*(*f*_*a *_| *f*_*j*_) is to take into account the association of two GO terms, *i.e. *the co-occurrence of the two GO terms in the same sequences. It is the conditional probability that *f*_*a *_is associated with *f*_*j*_. *c*(*f*_*a*_, *f*_*j*_) is number of times *f*_*a *_and *f*_*j *_are assigned simultaneously to each sequence in UniProt [57], and *c*(*f*_*j*_) is the total number of times *f*_*j *_appeared in UniProt, *μ *is the total number of unique GO terms considered in the associations, and *ε *is the pseudo-count, which is set to 0.05. Note that the conditional probability is asymmetric, *i.e. P*(*f*_*a *_| *f*_*j*_) ≠ *P*(*f*_*j *_| *f*_*a*_).

For running PSI-BLAST, the default E-value threshold for inclusion in multiple iterations (-h 0.005) is used and the maximum number of iterations is set to three (-j 3). By shifting the scoring space by a constant (*b*), individual annotations from weakly similar sequences (E-value > 1) can be considered and scored. Here we use *b *= *log*(125) to allow the use of sequence matches to an E-value of 125.

We also employed the score propagation by considering hierarchical relationship of the GO terms. Each GO term in the GO hierarchy (a directed acyclic graph) follows the true path rule; that is, any gene associated with a GO term must also be associated with the ancestors of that term leading back to the ontology root. Following this rule, we score ancestors of any predicted GO term according to the number of genes associated to the predicted term relative to the ancestor term:(4)

where *s(f*_*p*_) is the score of the parent term *f*_*p*_. *N*_*c *_is the number of child GO term which belong to the parent term *f*_*p *_and *s(f*_*ci*_) is the score of a child term *f*_*ci*_. *c(f*_*ci*_) and *c(f*_*p*_) is the number of known genes which are annotated with function term *f*_*ci *_and *f*_*p*_, respectively. The final raw score of a GO term is given by summing up the score which is directly computed by Eqn. 2 and those from the ancestral score propagation by Eqn. 4.

Finally, for each predicted GO term, the p-value of the raw score is computed by using the term-specific raw score distribution obtained by running PFP on the benchmark dataset [14]. Then, the expected accuracy is assigned to the prediction by referring to the correlation of the p-value and the actual accuracy computed for each GO term (see 6 in our previous paper [14]).

### PPI network enrichment

To evaluate enrichment of annotations in the interaction network, we compared the number of fully (both interaction partners annotated) and partially (one of the interaction partners annotated) annotated interactions before and after application of PFP to unannotated proteins in the dataset (Fig. 2). We considered only GO predictions with high confidence for the node enrichment.

### Partitioning PPI subnetworks

We used a randomization approach to partition the PPI networks into significant subnetworks. Subnetworks were created from the original dataset using each protein as a centroid, and including all directly interacting proteins and the edges between them. The original dataset was then randomized 100 times, maintaining the number of interactions for each protein while changing specific interacting partners. For each subnetwork *i*, the connectivity coefficient (*c*_*i*_) was calculated as the ratio of edges (*g*_*i*_) to nodes (*n*_*i*_) in the interaction subnetwork:(5)

Statistical significance of the connectivity coefficient of each real subnetwork was calculated using Student's T statistic (*α *= 0.05):(6)

where *ν *is the average value of the connectivity coefficient for the set of all subnetworks of the same centroid, and *s *is the variance of the connectivity coefficient values for the same set. This method of determining statistically significant subnetworks was used by LaCount *et al. *[44] for the malaria interaction network.

### Functional similarity network

Our novel concept of the functional similarity network uses individual proteins as nodes and scored functional similarities between proteins as edges. We have used the Schlicker method for calculating the similarity score between two sets of GO terms that uses the structure and information content of nodes in the GO hierarchy[38]. Using this method, the similarity of two individual GO terms *c*_*1 *_and *c*_*2 *_is(7)

where *p(c) *is the annotation frequency of term c relative to the frequency of the ontology root, and *S(c*_*1*_, *c*_*2*_) is the set of common ancestor terms between terms *c*_*1 *_and *c*_*2*_. The similarity of two sets of terms,  and , of respective sizes *N *and *M *is calculated by constructing an all-by-all similarity matrix *S*_*ij*_.(8)

Row vectors compare the similarity of set *A *(protein 1) to set *B *(protein 2), while column vectors compare the similarity of set *B *(protein 2) to set *A *(protein 1).(9)(10)

To calculate an overall similarity score for the two term sets, we combined these two terms for each GO category:(11)

where *GOscore *is any of the three category scores (*MF-score*, *BP-score*, *CC-score*). We differentiate from the Schlicker method only to include cellular component similarity into the overall score, which is calculated as(12)

max(*GOscore*) is set to 1 (maximum possible *GOscore*) and the range of the *funSim *score is [0,1]. To construct the function similarity networks for each organism, we performed an all-by-all pairwise comparison to find the *funSim *and category *GOscore *values for each unique protein pair.

In the functional similarity networks, pairs with the GOScore or *funSim *score of 0.95 or higher are connected by edges. The networks are visualized with Cytoscape [58].

### Identifying significant genomic windows

To identify functionally similar regions of a genome, we used a sliding window approach. For each organism we used a unique window size (10 kb for *E. coli*, 30 kb for *P. falciparum*, 15 kb for *S. cerevisiae*) and a slide value equal to 1/5 the window size. The window sizes were determined such that the number of genes for both strands in any window averaged between eight and ten. Genes included in the window were taken from the plus and minus strands individually and also from both strands together. Windows for which the category GO score was above 0.7 or the *funSim *was above 0.49 were analyzed for overrepresentation of GO functional terms by the method described below.

### Identifying significantly overrepresented terms in groups of proteins

Functional analysis of the PPI subnetworks and the genome windows is performed by identifying overrepresented GO terms in the subset relative to the annotation set of the entire proteome. Overrepresented terms are found essentially by applying the hypergeometric distribution to all terms annotated to proteins in the cluster [59]. The probability of a GO term *X *being annotated to a protein in the cluster is computed by:(13)

where *k *is the number of proteins in the cluster annotated with *X*, *N *is the number of annotated proteins in the organism, *m *is the number of proteins in the organism annotated with *X*, and *n *is the number of annotated proteins in the cluster. To calculate a p-value for overrepresentation of a term, we use this probability for annotation of *k *or more proteins in the cluster:(14)

Because we are analyzing overrepresentation of several GO terms, we use the false discovery rate (FDR) correction for multiple hypothesis testing:(15)

where q is the number of unique GO terms annotated to proteins in the cluster.

The annotation gain for a subset of proteins is calculated as the percentage increase in the number of unique new statistically overrepresented annotations as compared to the number of previously known annotations.

## Availability

PFP is available as a web tool http://kiharalab.org/pfp and as a downloadable distribution as used in these analyses http://kiharalab.org/pfp/dist. In addition, the supplemental data including the function annotation by PFP to the three genomes and the PPI networks and networks statistics of the functional similarity networks and the PPI networks are available at our lab website http://kiharalab.org/func_network_suppl/.

## Authors' contributions

TH implemented the algorithms, conducted the experiments, and drafted the paper. MC analyzed the function prediction by PFP and the properties of the functional similarity networks. DK conceived of the study, participated in its design, coordination, and finalized the manuscript. All authors read and approved the final manuscript.
